# Therapierelevante Antibiotikaresistenzen im One-Health-Kontext

**DOI:** 10.1007/s00103-023-03713-4

**Published:** 2023-05-15

**Authors:** Guido Werner, Muna Abu Sin, Christina Bahrs, Sandra Brogden, Andrea T. Feßler, Stefan Hagel, Heike Kaspar, Robin Köck, Lothar Kreienbrock, Henrike Krüger-Haker, Frederike Maechler, Ines Noll, Mathias W. Pletz, Bernd-Alois Tenhagen, Stefan Schwarz, Birgit Walther, Martin Mielke

**Affiliations:** 1 Robert Koch Institut, Berlin, Deutschland; 2 Abt. Infektionskrankheiten, Fachgebiet Nosokomiale Infektionserreger und Antibiotikaresistenzen, Robert Koch-Institut, Außenstelle Wernigerode, Burgstr. 37, 38855 Wernigerode, Deutschland; 3 WHO Collaborating Centre for Antimicrobial Resistance, Consumption and Healthcare-Associated Infections, Berlin, Deutschland; 4 Institut für Infektionsmedizin und Krankenhaushygiene, Universitätsklinikum Jena, Jena, Deutschland; 5 Institut für Biometrie, Epidemiologie und Informationsverarbeitung, Stiftung Tierärztliche Hochschule Hannover, Hannover, Deutschland; 6 WHO Collaborating Centre for Research and Training for Health at the Human-Animal-Environment Interface, Hannover, Deutschland; 7 Institut für Mikrobiologie und Tierseuchen, Fachbereich Veterinärmedizin, Freie Universität Berlin, Berlin, Deutschland; 8 Tiermedizinisches Zentrum für Resistenzforschung (TZR), Fachbereich Veterinärmedizin, Freie Universität Berlin, Berlin, Deutschland; 9 Bundesamt für Verbraucherschutz und Lebensmittelsicherheit, Berlin, Deutschland; 10 Bereich Hygiene und Umweltmedizin, Universitätsmedizin Essen, Essen, Deutschland; 11 Institut für Hygiene, Universitätsklinikum Münster, Münster, Deutschland; 12 Institut für Hygiene und Umweltmedizin, Charité – Universitätsmedizin Berlin, Berlin, Deutschland; 13 Fachbereich Epidemiologie, Zoonosen und Antibiotikaresistenz, Abteilung Biologische Sicherheit, Bundesinstitut für Risikobewertung BfR, Berlin, Deutschland; 14 Fachgebiet Mikrobiologische Risiken, Abteilung Umwelthygiene, Umweltbundesamt, Berlin, Deutschland

**Keywords:** MRSA, ESBL, MRGN, Healthcare-assoziierte Infektionen, Krankenhaus, Tiermast, Colistin, MRSA, ESBL, Multidrug-resistant organisms (MDRO), Healthcare-associated infections, Hospital, Livestock, Colistin

## Abstract

„One Health“ bezeichnet ein Konzept, das die Gesundheit von Menschen, Tieren und der Umwelt miteinander verbindet. In Deutschland gibt es umfangreiche Daten zur Antibiotikaresistenz (AMR) und multiresistenten Erregern (MRE) in der Human- und Veterinärmedizin sowie aus Untersuchungen in verschiedenen Umweltkompartimenten (Boden, Wasser, Abwasser). Die Erhebung erfolgt nach unterschiedlichen Vorgaben und Standards, was den Vergleich von Daten erschwert. Ein Fokus auf humantherapeutisch wichtige AMR und MRE ist hilfreich, um eine gewisse Orientierung vorzugeben. Die meisten Daten liegen sektorübergreifend zu Methicillin-resistenten *Staphylococcus aureus* und multiresistenten Enterobacterales wie *Escherichia coli* und *Klebsiella pneumoniae* vor. Hier sind die Trends der Resistenzen heterogen. Der Einsatz von Antibiotika führt zur Selektion von MRE, was gut dokumentiert ist. Erfolge bei der Minimierung des Antibiotikaeinsatzes konnten in zurückliegenden Jahren für einzelne Sektoren dargestellt und z. T. mit Erfolgen in der Eindämmung von AMR und MRE korreliert werden (Rückgang MRSA in der Humanmedizin). Auch sektorspezifische Maßnahmen zur Senkung der Last durch MRE und AMR sind notwendig, da Resistenzprobleme nicht generell eine Verknüpfung mit anderen Sektoren aufweisen. Carbapenemresistenzen sind vor allem bei pathogenen Erregern vom Menschen nachweisbar. Colistinresistenzen kommen in verschiedenen Sektoren vor, zeigen aber dort jeweils verschiedene Mechanismen. Resistenzen gegen Reservesubstanzen wie Linezolid sind in Deutschland selten, sie zeigen aber einen konkreten One-Health-Bezug. Bestrebungen zur Harmonisierung von Methoden, z. B. im Bereich der antimikrobiellen Empfindlichkeitstestung und genombasierten Erreger- und AMR-Surveillance, sind ein wichtiger erster Schritt zu einer Vergleichbarkeit der verschiedenen Datenerhebungen.

## Abstract (en)

One Health refers to a concept that links human, animal, and environmental health. In Germany, there is extensive data on antibiotic resistance (AMR) and multidrug-resistant (micro)organisms (MDRO) in human and veterinary medicine, as well as from studies in various environmental compartments (soil, water, wastewater). All these activities are conducted according to different specifications and standards, which makes it difficult to compare data. A focus on AMR and MDRO of human therapeutic importance is helpful to provide some guidance. Most data are available across sectors on methicillin-resistant *Staphylococcus aureus* (MRSA) and multiresistant Enterobacterales such as *Escherichia coli *and* Klebsiella pneumoniae*. Here, the trends of resistance are heterogeneous. Antibiotic use leads to MRE selection, which is well documented. Success in minimizing antibiotic use has also been demonstrated in recent years in several sectors and could be correlated with success in containing AMR and MDRO (e.g., decrease in MRSA in human medicine). Sector-specific measures to reduce the burden of MDRO and AMR are also necessary, as not all resistance problems are linked to other sectors. Carbapenem resistance is still rare, but most apparent in human pathogens. Colistin resistance occurs in different sectors but shows different mechanisms in each. Resistance to antibiotics of last resort such as linezolid is rare in Germany, but shows a specific One Health correlation. Efforts to harmonize methods, for example in the field of antimicrobial susceptibility testing and genome-based pathogen and AMR surveillance, are an important first step towards a better comparability of the different data collections.

## Das Problem der Antibiotikaresistenz in der Humanmedizin

Die Entwicklung, Verfügbarkeit und breite Anwendung von Antibiotika (Antiinfektiva) haben der Medizin bis dahin nicht gekannte Möglichkeiten erschlossen, Infektionskrankheiten effektiv zu therapieren [1]. Die mit dem Einsatz von Antibiotika untrennbar verbundene Problematik der Resistenzentwicklung kann diesen Fortschritt allerdings relevant beeinträchtigen. Antibiotikaresistenzen werden dann klinisch relevant, wenn sie in Krankheitserregern auftreten und die Wirksamkeit von Standardtherapien (Mittel der 1. Wahl) bzw. die verfügbaren Therapieoptionen einschränken.

Die Weltgesundheitsversammlung hat 2015 einen Globalen Aktionsplan gegen Antibiotikaresistenzen verabschiedet, der 5 Zielbereiche definiert, die gemeinsam mit der Ernährungs- und Landwirtschaftsorganisation der Vereinten Nationen (FAO) und der Weltorganisation für Tiergesundheit (WOAH, früher: OIE) entwickelt wurden und bis heute als Vorlage für die Entwicklung und Fortschreibung von nationalen Aktionsplänen dienen.

Hierbei wurden Erreger identifiziert, die aufgrund besonderer Eigenschaften bzw. Kriterien eine besondere Herausforderung für die Medizin darstellen. Zu diesen Erregern zählen Carbapenem-resistente *Acinetobacter baumannii* und *Pseudomonas aeruginosa*, Carbapenem-resistente und 3.-Generation-Cephalosporin-resistente Enterobacterales sowie Vancomycin-resistente *Enterococcus faecium* (VRE) und Methicillin-resistente *Staphylococcus aureus* (MRSA), aber auch Clarithromycin-resistente *Helicobacter pylori* und Fluorchinolon-resistente *Campylobacter* spp., Ceftriaxon-resistente *Neisseria gonorrhoeae* und multiresistente *Salmonella *Typhi [2]. Diese Liste stellt eine Hilfe bei der Priorisierung von Maßnahmen dar, macht aber auch deutlich, dass eine weitergehende Betrachtung geboten ist, um den mit der Resistenz verbundenen, aktuellen Problemen in der klinischen Praxis gerecht zu werden.

Im Rahmen eines über die alleinig humanmedizinische Betrachtung hinausgehenden One-Health-Kontextes stellen sich hierbei Fragen wie: Welcher Anteil der Resistenzproblematik ist durch Maßnahmen im Rahmen der Humanmedizin allein zu beherrschen? Welcher Anteil humanmedizinisch relevanter Resistenzen kann nur durch Maßnahmen in anderen Bereichen beherrscht werden? Diesen und weiteren assoziierten Fragen geht der Artikel in den folgenden Abschnitten vor allem aus einem One-Health-Blickwinkel nach.

Bei klassischen zoonotischen Erregern ist der Zusammenhang zwischen dem Einsatz von Antibiotika beim Tier und möglichen Resistenzen bei Erregern, die zur Infektion des Menschen führen, offensichtlich (etwa Resistenzen bei *Campylobacter* spp. und *Salmonella*). Resistenzprobleme bei *Neisseria gonorrhoeae *und* Helicobacter pylori *als klassische, ausschließlich humanpathogene Erreger sind ganz überwiegend auf dem Einsatz von Antibiotika in der Humanmedizin begründet. Diese Themen stehen bewusst nicht im Fokus der Ausführungen in diesem Artikel.

## Hochauflösende Analysen von Resistenzmechanismen und ihre Bedeutung für den Therapieerfolg

Entwicklungen der letzten Jahre haben die molekularen Mechanismen vieler Resistenzphänomene aufgedeckt. Beim hochauflösenden Nachweis von Resistenzmechanismen muss zwischen der molekularen Analyse eines kulturell nachgewiesenen Isolates und den PCR-basierten direkten, kulturunabhängigen Nachweisen von Resistenzgenen direkt aus Patientenmaterial (z. B. Blut) unterschieden werden. Der direkte Nachweis von Resistenzgenen aus klinischem Material generiert speziesspezifische Unsicherheiten in der Interpretation:

Grampositive Bakterien: Bei MRSA und VRE determiniert ein singuläres Resistenzgen (MRSA: *mecA* oder *mecC*, VRE: meist durch *vanA* oder *vanB*) den Resistenzphänotyp einer generellen Beta-Laktam- bzw. Vancomycin-Resistenz [3]. Die Übereinstimmung des mittels molekularbiologischer Verfahren bestimmten Genotyps mit dem Phänotyp ist gegeben und eine entsprechende gezielte Therapie kann sogar allein aufgrund des genotypischen Nachweises der entsprechenden Resistenzgene begonnen werden.

Bei multiresistenten gramnegativen Bakterien (MRGN) ist die Situation komplexer, da die Resistenz gegen Beta-Laktame aus einer Kombination verschiedener Beta-Laktamasen – mit jeweils unterschiedlichen Expressionsniveaus – mit Porinverlust und Effluxpumpen generiert wird [4, 5]. Die Genotyp-Phänotyp-Korrelation ist hier deutlich unsicherer als bei MRSA und VRE. So kann eine Carbapenemresistenz durch Carbapenemasen (z. B. regelhaft bei *A. baumannii*) oder die Kombination von Porinverlust und AmpC (Beta-Laktamase-kodierendes Gen) vermittelt werden. Da die meisten neuen Beta-Laktam-Reserve-Antibiotika (s. unten) nur bestimmte, aber nicht alle Carbapenemresistenz-Mechanismen überwinden, sind sowohl eine phänotypische als auch eine genotypische Analyse der Carbapenemresistenz und ein zielgenauer Einsatz (s. unten) erforderlich, um ein Therapieversagen zu vermeiden. Dennoch ermöglicht der schnelle Nachweis einer Carbapenemase oder ihres Gens direkt aus der Blutkultur (ohne das Anlegen einer Kultur) eine frühzeitige, gezielte Anpassung der Antibiotikatherapie und verbessert damit den Behandlungserfolg.

## Therapieoptionen bei nachgewiesenen Antibiotikaresistenzen

MRSA-Infektionen können durch viele alte und neue Antibiotika therapiert werden. Neben Vancomycin, das lange Jahre als Referenzsubstanz bei MRSA galt, stehen heute Linezolid, Daptomycin, die MRSA-wirksamen Cephalosporine Ceftarolin und Ceftobiprol, Lipoglycopeptide wie Dalbavancin und das Glycylcyclin Tigecyclin zur Verfügung. Aber auch „alte“ Antibiotika wie Trimethoprim/Sulfamethoxazol und Doxycyclin sind oftmals noch wirksam [6].

Bei VRE hängt es vom Resistenzmechanismus ab: Bei *vanB* sind das Glycopeptid Teicoplanin und das Lipoglycopeptid Dalbavancin noch wirksam, bei Vorliegen von *vanA* wirkt kein Glycopeptid mehr, aber das Lipoglycopeptid Oritavancin ist noch wirksam. Tigecyclin kommt primär bei intraabdominalen VRE-Infektionen zum Einsatz.

Bei schweren MRGN-Infektionen mit Carbapenemresistenz war über Jahre das nephrotoxische Colistin ggf. in Kombination mit etwa Tigecyclin oder Fosfomycin eine favorisierte Therapie [7]. Mit der Entwicklung neuer Beta-Laktam-Antibiotika stehen jedoch nun bessere und weniger nephrotoxische Alternativen, wie z. B. Imipenem/Relebactam und Meropenem/Varbobactam, Ceftazidim/Avibactam und Cefiderocol, für die Therapie zur Verfügung. Aufgrund dessen empfiehlt z. B. die Infectious Diseases Society of America (IDSA) primär diese neuen Antibiotika einzusetzen und Colistin zu meiden [8]. Bis auf Cefiderocol haben jedoch alle diese neuen Antibiotika eine Wirksamkeitslücke gegenüber bestimmten Carbapenemresistenzmechanismen, insbesondere bei den Metallo-Beta-Laktamasen [9].

Zusammenfassend ist festzustellen, dass die klinische Relevanz bzw. die Therapieoptionen nicht nur vom Erreger, sondern auch vom Infektionsfokus und den Begleiterkrankungen beim Patienten bestimmt und ggf. eingeschränkt werden. Insbesondere bei MRGN ist für eine erfolgreiche und gezielte Therapie außerdem die Kenntnis des zugrunde liegenden Resistenzmechanismus für die Auswahl der optimalen Therapie erforderlich.

## Zusammenhang zwischen Besiedlung bzw. Kolonisierung und einer später auftretenden Infektion

Der Kontakt mit antibiotikaresistenten Bakterien erfolgt in der Regel zunächst über die Haut bzw. die Schleimhäute und führt anschließend ggf. zu einer Kolonisation z. B. des Darmtraktes nach oraler Aufnahme mit entsprechend kontaminierter Nahrung. In vielen Studien ist der Zusammenhang einer bakteriellen Besiedlung und einer späteren Infektion unter bestimmten Prädispositionen (Begleitumständen) hinlänglich untersucht, ausgewertet und beschrieben worden. Bei den meisten dieser Studien lag der Fokus nicht zwangsläufig auf (multi)resistenten Erregern, die Zusammenhänge sind aber auf Vertreter dieser entsprechenden Gattungen und Spezies übertragbar, wenn sie zusätzliche Resistenzen besitzen. Sowohl bei Kolonisation der Haut und der Schleimhäute wie bei Staphylokokken (*S. aureus*) als auch bei Kommensalen der Darmflora wie *E. coli* und *K. pneumoniae* ist dieser Zusammenhang dokumentiert [10, 11].

Ein enger physischer Kontakt ist ein Risikofaktor für eine Übertragung von Bakterien, was neben klassischen Situationen in Versorgungs- und Pflegeeinrichtungen (Krankenhäuser, Alten- und Pflegeheime) auch auf das familiäre Umfeld („Mutter-Kind-Transmissionen“; [12]) und auf Mitarbeitende in der Nutztierhaltung zutrifft (Nutztier-Tierpfleger/Tierarzt-Transmissionen; [13, 14]).

Eine klonale (vertikale) Verbreitung von resistenten Bakterien innerhalb von Sektoren oder aber auch darüber hinaus lässt sich durch einen Nachweis verwandter Isolate rekonstruieren. Eine Reihe von Antibiotikaresistenzen verbreitet sich aber auch horizontal, d. h. zwischen nichtverwandten Bakterien, z. T. auch zwischen Vertretern verschiedener Spezies und Gattungen. Insofern ist ein direkter Zusammenhang im Rahmen von Ausbruchsanalysen oder der Nachverfolgung von Transmissionsereignissen deutlich schwieriger zu rekonstruieren. Die Ableitung eines möglichen Zusammenhangs kann in diesen Fällen nur über hochauflösende Analyseverfahren wie moderne Sequenzier- und Auswertetechniken erfolgen [15, 16]. Somit gelingt die Rekonstruktion der Ausbreitung von Antibiotikaresistenzen in sogenannten Multispezies-Ausbrüchen [17, 18] als auch der Nachweis einer Verbreitung resistenter Erreger und ihrer Resistenzgene mittels bestimmter Vektoren (mobile bzw. übertragbare, genetische Elemente wie Plasmide und/oder Phagen) über verschiedene Sektoren im Bereich Tier-Lebensmittel-Mensch [16, 19].

## Surveillance der Antibiotikaresistenz im Bereich der Humanmedizin

Mit der Antibiotika-Resistenz-Surveillance (ARS; https://ars.rki.de) existiert in Deutschland eine Infrastruktur, die aktuelle Analysen zur Resistenzsituation und -entwicklung bei humanpathogenen Erregern ermöglicht. Labore, die Proben aus medizinischen Versorgungseinrichtungen mikrobiologisch untersuchen, übermitteln auf freiwilliger Basis die Ergebnisse von Identifizierung und Empfindlichkeitsprüfung aller humanpathogenen Erreger aus allen Probenmaterialien elektronisch an das Robert Koch-Institut (RKI).

Nachfolgend werden Ergebnisse für den Zeitraum 2017–2021 präsentiert; sie basieren auf Daten von Krankenhäusern und Arztpraxen, die für den gesamten Zeitraum vollständige Daten übermittelt haben (*N* = 497 Krankenhäuser, die ca. 27 % der allgemeinen Krankenhäuser in Deutschland repräsentieren, sowie 10.272 Arztpraxen, was einer Abdeckung (Coverage) von 13 % entspricht). In die Berechnung der phänotypischen Resistenzrate gehen nur Erstisolate pro Spezies, Patient, Jahr und ggf. Probenmaterial ein.

Die Ergebnisse sind in Tab. 1 zusammengefasst. Für die Cephalosporine der 3. Generation ergeben sich statistisch signifikant (*p* < 0,05) abnehmende Trends für *E. coli* und *K. pneumoniae* in allen untersuchten Segmenten. Die Ergebnisse zur Carbapenemresistenz sind heterogen: Bei *K. pneumoniae *sind keine signifikanten Trends zu beobachten. Für *P. aeruginosa* finden sich signifikant steigende Resistenzanteile sowohl in der stationären als auch in der ambulanten Versorgung, wohingegen für *A. baumannii complex* ein abnehmender Trend in der stationären Versorgung zu verzeichnen ist.

**Table 1:** 

Spezies	Antibiotikum	Sektor	Material	2017	2018	2019	2020	2021	Trend 2017–2021
				%	%	%	%	%	
*E. coli*	Ceph. 3. Gen.	Stationär	Alle	11,1	11,1	10,8	9,7	8,6	**↓**
*E. coli*	Ceph. 3. Gen.	Stationär	Blut	12,6	12,3	11,8	10,3	9,0	**↓**
*E. coli*	Ceph. 3. Gen.	Stationär	Urin	11,0	11,0	10,7	9,5	8,5	**↓**
*E. coli*	Ceph. 3. Gen.	Ambulant	Alle	6,4	7,1	7,1	6,2	5,4	**↓**
*E. coli*	Ceph. 3. Gen.	Ambulant	Urin	6,4	7,1	7,2	6,2	5,5	**↓**
*K. pneumoniae*	Ceph. 3. Gen.	Stationär	Alle	12,3	12,2	11,1	10,3	9,3	**↓**
*K. pneumoniae*	Ceph. 3. Gen.	Stationär	Blut	14,5	13,5	11,9	11,1	10,2	**↓**
*K. pneumoniae*	Ceph. 3. Gen.	Stationär	Urin	12,4	12,7	11,2	10,5	9,6	**↓**
*K. pneumoniae*	Ceph. 3. Gen.	Ambulant	Alle	7,4	7,3	7,3	6,6	6,0	**↓**
*K. pneumoniae*	Ceph. 3. Gen.	Ambulant	Urin	7,5	7,3	7,3	6,9	6,0	**↓**
*K. pneumoniae*	Carbapeneme	Stationär	Alle	0,4	0,4	0,4	0,4	0,4	**–**
*K. pneumoniae*	Carbapeneme	Stationär	Blut	0,76	0,63	0,91	0,52	0,78	**–**
*K. pneumoniae*	Carbapeneme	Stationär	Urin	0,25	0,33	0,30	0,25	0,29	**–**
*K. pneumoniae*	Carbapeneme	Ambulant	Alle	0,06	0,10	0,10	0,09	0,05	**–**
*K. pneumoniae*	Carbapeneme	Ambulant	Urin	0,02	0,03	0,09	0,06	0,04	**–**
*P. aeruginosa*	Ceftazidim	Stationär	Alle	8,2	8,3	8,8	8,9	8,2	**–**
*P. aeruginosa*	Ceftazidim	Stationär	Blut	10,1	9,3	9,1	9,6	9,8	**–**
*P. aeruginosa*	Ceftazidim	Ambulant	Alle	3,9	3,8	4,4	4,0	3,9	**–**
*P. aeruginosa*	Carbapeneme	Stationär	Alle	12,1	11,5	12,1	12,8	12,7	**↑**
*P. aeruginosa*	Carbapeneme	Stationär	Blut	12,6	12,0	12,1	13,8	14,2	**–**
*P. aeruginosa*	Carbapeneme	Ambulant	Alle	7,2	7,1	8,3	8,3	8,0	**↑**
*A. baumannii co.*	Carbapeneme	Stationär	Alle	3,6	3,3	3,3	2,7	2,6	**↓**
*A. baumannii co.*	Carbapeneme	Stationär	Blut	6,6	5,6	3,6	6,9	10,2	**–**
*A. baumannii co.*	Carbapeneme	Ambulant	Alle	1,4	1,3	1,1	0,7	0,7	**–**
*S. aureus*	Oxacillin	Stationär	Alle	11,4	10,5	9,1	7,5	6,6	**↓**
*S. aureus*	Oxacillin	Stationär	Blut	9,3	8,1	6,8	5,4	4,9	**↓**
*S. aureus*	Oxacillin	Stationär	Wunde	10,0	9,2	8,1	6,9	5,9	**↓**
*S. aureus*	Oxacillin	Ambulant	Alle	7,0	6,7	5,6	4,9	4,3	**↓**
*S. aureus*	Oxacillin	Ambulant	Wunde	8,1	7,4	6,4	5,5	4,7	**↓**
*E. faecium*	Vancomycin	Stationär	Blut	17,0	23,0	25,0	21,9	20,0	**–**
*E. faecium*	Teicoplanin	Stationär	Blut	8,6	8,2	9,9	10,6	8,8	**–**

Trends wurden mit einem Chi^2^-Test for Trends getestet

*Ceph. 3. Gen.* Cephalosporine der 3. Generation: Cefotaxim/Ceftriaxon/Ceftazidim, *Carbapeneme* Imipenem/Meropenem, *Oxacillin* steht hier synonym für „MRSA“ (Methicillin-resistente(r) *Staphylococcus aureus*): Cefoxitin oder Oxacillin/Flucloxacillin, *co.* complex

Bei MRSA setzt sich der seit Jahren belegte signifikante Rückgang in Blutkulturen fort und ist auch in der stationären wie ambulanten Versorgung allgemein sowie bei Isolaten aus Wundmaterial zu verzeichnen (Tab. 1). Der Rückgang von MRSA in invasiven Isolaten (Isolate aus Blut/Liquor) zeigt sich auch deutlich in den Daten aus der Meldepflicht, die ausgehend von 3602 Meldungen im Jahr 2015 in den zurückliegenden Jahren bis auf 1126 Fälle im Jahr 2020 jährlich rückläufig sind (2016: 3136; 2017: 2835; 2018: 2424; 2019: 1810; s. Infektionsepidemiologische Jahrbücher 2015–2020, nach Referenzdefinitionen C + D + E).^1^ Mit Beginn der Coronapandemie stagnierten die Meldungen von Enterobacterales-Infektionen oder -Kolonisationen mit verminderter Empfindlichkeit gegenüber Carbapenemen, nachdem sie in den Vorjahren seit Einführung der Meldepflicht beständig angestiegen waren (2017: 2577; 2018: 4041; 2019: 4683; 2020: 3533; s. Infektionsepidemiologische Jahrbücher 2017–2020, nach Referenzdefinitionen C + D + E). Der ebenfalls seit einigen Jahren anhaltende Trend zur Zunahme von VRE bei Isolaten aus Blutkulturen scheint sich zwar nicht fortzusetzen; allerdings steigt hier die Anzahl der Nachweise von *E. faecium* aus Blutkulturen, wodurch absolut mehr VRE zu verzeichnen sind.

Daten zu Empfindlichkeiten gegenüber Reservesubstanzen wie Colistin, Cefiderocol und den neuen Beta-Laktam/Beta-Laktamaseinhibitor-Kombinationen liegen vor allem in Form von Studien und Sentinelerhebungen vor [20–22].

## Surveillance multiresistenter Erreger auf deutschen Intensivstationen

Bei den verschiedenen Erregergruppen zeigte sich für Deutschland in den letzten durch die Pandemie geprägten Jahren in den Intensivstationen eine heterogene Entwicklung, die sich deutlich von anderen Ländern unterscheidet [23].

Die Daten entstammen der Erregersurveillance in Intensivstationen aus dem Krankenhaus-Infektions-Surveillance-System KISS am Nationalen Referenzzentrum für Nosokomiale Infektionen (NRZ – https://www.nrz-hygiene.de/das-nrz). Die Erregersurveillance im KISS basiert auf der freiwilligen Teilnahme von Krankenhäusern, die Ergebnisse zu Erregeridentifikation und Resistenztestung aus der klinischen Routinediagnostik an das NRZ übermitteln. In die Dokumentation gehen sowohl Besiedlungen als auch Infektionen mit den jeweiligen Erregern ein, die im Folgenden nicht unterschieden werden. Die für 5‑Jahres-Zeiträume berechneten Referenzdaten unter Berücksichtigung der einzelnen Erreger, der Stationsart und der Screeningintensität können auf der Webseite abgerufen werden.^2^

Im Jahr 2021 übermittelten 393 Intensivstationen Daten zur Erregersurveillance an das NRZ. Damit war die Anzahl teilnehmender Intensivstationen im Vergleich mit den vorherigen Jahren konstant, wobei die Anzahl der in den teilnehmenden Stationen intensivmedizinisch versorgten Patienten während der Pandemie rückläufig war (2019: 409.146; 2021: 314.161, Reduktion um 23 %). Im selben Zeitraum verlängerte sich die Liegezeit der Patienten von durchschnittlich 4,0 auf 4,4 Tage.

Für die heterogene Gruppe der multiresistenten gramnegativen Bakterien (MRGN) konnte insgesamt nach einem Anstieg der Besiedlungsraten bis 2019 in den Pandemiejahren ein Rückgang beobachtet werden. Diese Entwicklung zeigte sich insbesondere bei 3MRGN^3^*E. coli* und 3MRGN* K. pneumoniae *(Rückgang der Aufnahmeprävalenz um 18 % bei *E. coli* und um 17 % bei *K. pneumoniae*, Tab. 2).

**Table 2:** 

MRE	Spezies	Jahr	Aufnahmeprävalenz (%)	Trend	*p*-Wert^a^	Inzidenz	Trend	*p*-Wert^a^
**3MRGN**	*E. coli*	2019	1,528	**↓**	<0,01	0,185	–	0,30
		2020	1,425			0,170		
		2021	1,253			0,197		
	*K. pneumoniae*	2019	0,369	**↓**	<0,01	0,087	–	0,16
		2020	0,334			0,071		
		2021	0,304			0,075		
**4MRGN**	*A. baumannii*	2019	0,027	–	0,14	0,006	–	0,27
		2020	0,024			0,009		
		2021	0,022			0,008		
	*E. coli*	2019	0,019	–	0,50	0,005	**↑**	0,04
		2020	0,017			0,008		
		2021	0,021			0,009		
	*K. pneumoniae*	2019	0,044	–	0,86	0,021	–	0,55
		2020	0,037			0,021		
		2021	0,044			0,023		
	*P. aeruginosa*	2019	0,121	–	0,93	0,059	**↑**	0,01
		2020	0,115			0,060		
		2021	0,121			0,074		
**MRSA**	*S. aureus*	2019	0,975	**↓**	<0,01	0,105	**↓**	<0,01
		2020	0,849			0,091		
		2021	0,731			0,084		
**VRE**	*E. faecium*	2019	1,501	–	0,14	0,489	**↑**	<0,01
		2020	1,547			0,477		
		2021	1,541			0,574		

Die dargestellten Daten umfassen Kolonisationen und Infektionen mit MRE aus der klinischen Routine ohne Berücksichtigung von Screeningmaßnahmen

*Aufnahmeprävalenz* aufgenommene Patienten mit MRE/100 Patienten, *Inzidenz* Patienten mit während des stationären Aufenthaltes neu festgestellten MRE/100 Patienten, *3MRGN* Resistenz gegen Piperazillin, Ciprofloxacin und einem 3. Generation-Cephalosporin, *4MRGN* 3MRGN und zusätzliche Carbapenemresistenz, *MRE* multiresistente(r) Erreger, *MRSA* Methicillin-resistente(r) *Staphylococcus aureus, VRE* Vancomycin-resistente(r) *Enterococcus (faecium)*

^a^Cochrane Armitage Test für Trends

Im Gegensatz dazu zeigte sich bei den 4MRGN^4^ auf einem insgesamt im internationalen Vergleich noch sehr niedrigen Niveau ein Anstieg von im Krankenhaus neu festgestellten 4MRGN *P. aeruginosa* und der 4MRGN *E. coli *(relative Zunahme der Inzidenz um 26 % bei *P. aeruginosa* bzw. um 84 % bei *E. coli* seit 2019, Tab. 2). Im selben Zeitraum wurde im Rahmen der Antibiotika-Verbrauchssurveillance AVS am RKI zwar nur eine geringe relative Zunahme im Gesamtverbrauch von Antibiotika in Intensivstationen festgestellt (Median 95,93 auf 100,83 DDD^5^/100 Patiententage von 2019 bis 2021), der Carbapenemverbrauch stieg allerdings um ca. 17 % an (Median 11,64 auf 14,03 DDD/100 Patiententage von 2019 bis 2021).^6^

Bei den VRE hat sich seit dem deutlichen Anstieg bis 2018 die weitere Zunahme während der Pandemie verlangsamt (Zunahme der Inzidenz um 17 %, Tab. 2). Dagegen setzte sich der bereits seit mehreren Jahren beschriebene rückläufige Trend für den MRSA unvermindert fort.

## Resistenzmonitoring bei Nutztieren und Lebensmitteln sowie Antibiotika-Verbrauchssurveillance im Nutztierbereich

Viele Antibiotika werden sowohl bei Tieren als auch bei Menschen angewendet. Durch die Änderungen im europäischen Tierarzneimittelrecht ist nunmehr rechtlich geregelt, welche Antibiotika bei Tieren nicht eingesetzt werden dürfen [24]. Zuvor bestand diese Einschränkung v. a. für den Einsatz bei lebensmittelliefernden Tieren. Substanzen für die keine Rückstandshöchstgrenze („maximum residue limit“) definiert war, waren vom Einsatz grundsätzlich ausgeschlossen. Nicht ausgeschlossen sind allerdings einige Substanzen, die von der Weltgesundheitsorganisation (WHO) als „highest priority critically important antimicrobials“ (hpCIA) klassifiziert werden, darunter die Cephalosporine der 3. und 4. Generation, die Fluorchinolone, die Polymyxine (z. B. Colistin). Alle diese Substanzklassen wurden und werden bei lebensmittelliefernden Tieren eingesetzt und entsprechend werden auch bei Bakterien von Tieren und den von ihnen stammenden Lebensmitteln Resistenzen gegen diese Substanzen gefunden. Besonders häufig ist dies bei Fluorchinolonen der Fall, bei denen u. a. Enterobacterales aus Geflügelfleisch, insbesondere Hähnchenfleisch, häufig erworbene Resistenzen aufweisen [25, 26].

Seit dem Jahr 2011 werden in Deutschland jährlich die Mengen der an Tierärzte verkauften Antibiotika erhoben. Insgesamt ist der Verkauf von „highest priority critically important antimicrobials“ (hpCIA) in der Tierhaltung in den letzten Jahren (2011–2021) deutlich zurückgegangen [27]. Für die Fluorchinolone (−32,5 %) und Cephalosporine der 3. und 4. Generation (−66,2 %) ist dies neben den Dokumentationspflichten nach Arzneimittelgesetz seit 2014 mit erhöhten diagnostischen Anforderungen und dem Verbot der Umwidmung erklärbar, die mit der Änderung der tierärztlichen Hausapothekenverordnung im Jahr 2018 eingeführt wurden [28]. Für Colistin (−59,7 %) entspricht es zudem dem von der Europäischen Arzneimittelbehörde EMA geforderten Rückgang des Einsatzes dieser Substanzen [27].

Der Einsatz der verschiedenen Substanzklassen und auch die absolute Therapiehäufigkeit für alle Substanzen unterscheiden sich zwischen den verschiedenen Tierarten. Während bei Masthähnchen häufig Colistin und Fluorchinolone eingesetzt werden, sind Cephalosporine der 3. und 4. Generation hier nicht zugelassen. Beim Schwein ist der Einsatz von Fluorchinolonen und Cephalosporinen gering, der von Colistin, insbesondere bei Ferkeln nach der Trennung von der Sau, höher. Beim Rind liegen im Rahmen des Antibiotikaminimierungskonzeptes nur Daten zu den Mastbereichen vor, nicht aber zu Milchkühen. Während in den Mastbereichen der Einsatz von Cephalosporinen gering ist, ist dieser beim Milchrind wesentlich höher [29].

Resistenzdaten aus Tierhaltung und Lebensmittelproduktion werden in Europa auf der Grundlage eines Durchführungsbeschlusses der EU-Kommission ermittelt. In Deutschland werden die Programme jährlich durch weitere Programme ergänzt. Seit dem Jahr 2001 trägt das Bundesamt für Verbraucherschutz und Lebensmittelsicherheit darüber hinaus Daten zur Empfindlichkeit von Bakterien zusammen, die von erkrankten Nutztieren isoliert wurden. Unter den hpCIA werden Resistenzen bei Isolaten von Tieren und Lebensmitteln besonders häufig gegenüber Fluorchinolonen beobachtet. Isolate von Enterobacterales aus Geflügelfleisch, insbesondere Hähnchenfleisch, weisen häufig erworbene Resistenzen auf. Die Nachweisraten von Resistenzen gegenüber Fluorchinolonen bei *E.-coli-*Isolaten von Schwein und Rind und ihrem Fleisch sind dagegen substanziell geringer (Tab. 3). Hinzu kommt, dass die Übertragung der Bakterien von den Tieren auf die Schlachtkörper, bedingt durch unterschiedliche Schlachttechniken, bei Rind und Schwein in wesentlich geringerem Umfang stattfindet als beim Geflügel. Dadurch ist die Belastung von Geflügelfleisch aus beiden Gründen – höhere Resistenzraten und häufigere Übertragung – deutlich höher als die Belastung von Rind- und Schweinefleisch.

**Table 3:** 

Tierart, Anzahl untersuchte Isolate		Cefotaxim	Ceftazidim	Ciprofloxacin	Nalidixinsäure
Pute, *n* = 660	*n*	31	27	267	218
	%	4,7	4,1	40,5	33
Hähnchen, *n* = 1004	*n*	54	51	461	403
	%	5,4	5,1	45,9	40,1
Schwein; *n* = 220	*n*	5	5	11	6
	%	2,3	2,3	5,0	2,7
Kalb, *n* = 70	*n*	3	3	9	9
	%	4,3	4,3	12,9	12,9
Rind, *n* = 333	*n*	4	3	7	7
	%	1,2	0,9	2,1	2,1

Während Cephalosporine der 3. und 4. Generation und Fluorchinolone beim Menschen in wesentlich größerem Umfang eingesetzt werden als bei lebensmittelliefernden Tieren und damit in beiden Bereichen eine umfangreiche Resistenzselektion stattfindet, verhält sich die Situation bei den Polymyxinen, konkret dem Colistin, anders. Dieses wird in der Humanmedizin aufgrund der häufigen Nebenwirkungen nur sehr selten und nur im stationären Bereich eingesetzt (s. oben), während es in der Tierhaltung häufig als oral verabreichte Bestandsmedikation im Rahmen der Therapie von Durchfallerkrankungen breit eingesetzt wird [29]. Entsprechend häufig ist seit Jahren der Nachweis von Colistinresistenzgenen *mcr* (*mcr1–mcr10*), die auf mobilen, genetischen Elementen angesiedelt sind [30, 31]. Eine Resistenz gegenüber Colistin bei *E.-coli-*Isolaten von gesunden und erkrankten Nutztieren ist entweder bedingt durch Punktmutationen auf den chromosomal kodierten Genen *pmrA*/*B* oder vermittelt durch ein plasmidkodiertes *mcr*-Gen. In einer retrospektiven Untersuchung konnte gezeigt werden, dass chromosomale Mutationen im Laufe der Studienjahre an Bedeutung verlieren und sich die plasmidvermittelte Übertragung von *mcr*-Genen durchsetzt. Der Anteil der *mcr*-tragenden Isolate an den Colistin-resistenten *E.-coli-*Isolaten von erkrankten Tieren war beim Schwein am höchsten (73 %), gefolgt von Geflügelisolaten (18 %) und Isolaten vom Rind (9 %). Im Studienjahr 2020 wurde die Variante *mcr-*1 am häufigsten gefunden (73 %), gefolgt von *mcr*-4 (14 %) und *mcr*-5 (15 %). Die Sequenztypen ST1, ST10 und ST42 waren dabei dominant vertreten, wohingegen der in der Humanmedizin wichtigste Erreger *E. coli* ST131 (> 2 %) nur sehr selten bei Haustier- und Nutzgeflügelisolaten gefunden wurde. Insgesamt war der Anteil von Isolaten mit minimalen Hemmstoffkonzentrationen (MHK) von Colistin > 2 mg/L mit weniger als 3 % relativ gering.

**Figure 1:**
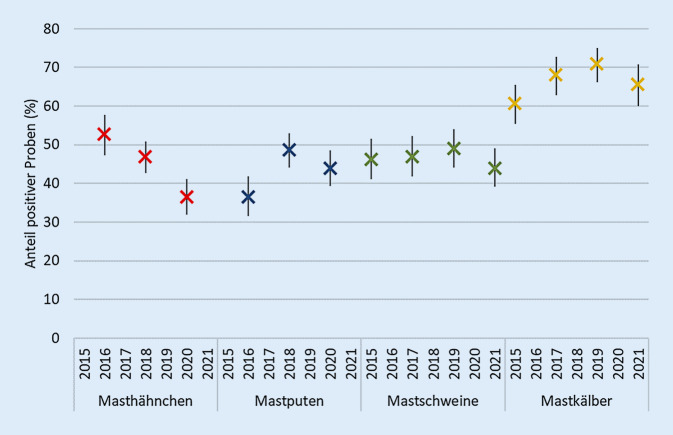

Die Ergebnisse der selektiven Untersuchung auf Cefotaxim-resistente *E. coli* in Blinddarminhalt von Schlachttieren zeigt bei den verschiedenen Tierarten unterschiedliche Entwicklungen. Während es bei Masthähnchen zu einem deutlichen Rückgang der Nachweise kam (2016: 52,6 %; 2020: 36,5 %), stiegen die Nachweisraten bei Schlachtschweinen, Schlachtputen sowie Mastkälbern und Jungrindern bis 2018 bzw. 2019 an, um danach leicht zurückzugehen (Abb. 1).

Meropenem-resistente *E. coli* wurden sporadisch in Kotproben von Schweinen oder im Schweinefleisch festgestellt. Der letzte Nachweis bei Monitoringuntersuchungen in Deutschland erfolgte 2019 [32–34]. Carbapeneme waren für den Einsatz bei Nutztieren nie zugelassen und es wurde die Hypothese geäußert, dass das sporadische Auftreten dieser Resistenz auf eine Übertragung vom Menschen auf das Tier zurückgeht [32, 35], wobei es vereinzelt auch zur Etablierung solcher Resistenzen in Tierpopulationen kommen kann [35]. Im Nationalen Resistenzmonitoring tierpathogener Erreger im Nutztierbereich werden die eingegangenen Enterobacterales routinemäßig auf ihr Resistenzverhalten gegenüber Imipenem untersucht, um Hinweise auf eine Carbapenemresistenz zu erkennen. Bisher wurden hierbei jedoch keine resistenten Isolate gefunden [36].

Enterokokkenisolate (*E. faecium* und *E. faecalis*) von Schlachttieren werden in Deutschland im Monitoring seit 2015 auf ihre Resistenz gegenüber Vancomycin untersucht. Keines der untersuchten 2002 Isolate aus Masthähnchen, Mastputen, Mastschweinen und Mastkälbern/Jungrindern am Schlachthof war resistent gegen Vancomycin oder Linezolid.

**Figure 2:**
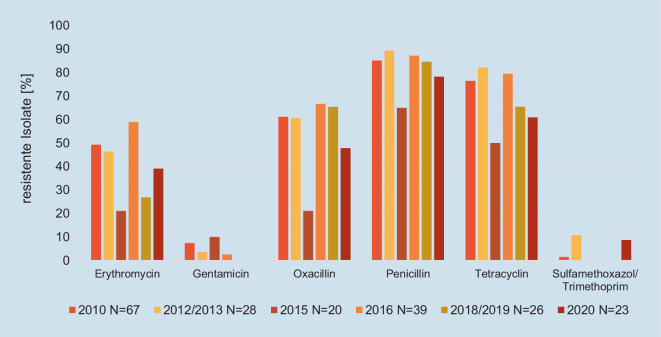

MRSA werden bei allen Nutztierspezies nachgewiesen, besonders häufig bei Schweinen, Puten und Mastkälbern. Meist treten sie nur als Besiedler auf, ohne dass die Nutztiere erkranken. Ausnahmen stellen Entzündungen der Milchdrüse des Rindes und Hautinfektionen beim Schwein dar. Von den untersuchten *S.-aureus*-Isolaten aus Hautinfektionen beim Schwein erwiesen sich 48 % als MRSA mit nachgewiesenem *mecA*-Gen (Abb. 2). Sie gehörten zum überwiegenden Teil dem Nutztier-assoziierten Sequenztyp (ST) 398 an. Dieser Sequenztyp mit zoonotischem Potenzial gehört zu denen, die sowohl beim Menschen als auch beim Nutztier gefunden werden können [36]. Bei den untersuchten MRSA wurde nur sehr selten eine Vancomycin-Resistenz festgestellt (6/3174 Isolaten = 0,2 % aus den Lebensmittelketten Huhn, Pute, Rind und Schwein; Daten aggregiert aus dem Zoonosen-Monitoring 2012–2021). Eine erhöhte Prävalenz für die Besiedlung des Menschen zeigt sich deutlich bei Beschäftigten in schweinehaltenden Betrieben und auch bei Tierärzten, die in einer auf Schweine spezialisierten Praxis tätig sind (siehe unten).

## Multiresistente Erreger (MRE) in Tierkliniken in Deutschland

Zahlreiche Berichte über Infektionen mit nosokomialen Infektionserregern aus Kliniken für kleine Haustiere und Pferde zeigen, dass MRE in Tierkliniken in einem steigenden Umfang nachgewiesen werden (Übersicht in [37]). Vor allem postoperative Wundinfektionen, Harnwegsinfektionen, Blutstrominfektionen und infektiöse Diarrhöen werden beschrieben [37]. Anders als in der Humanmedizin gibt es keinerlei gesetzliche Regelungen über eine Aufzeichnungs- oder Meldepflicht, so dass Zahlen zu MRE aus diesem Bereich nur exemplarisch vorliegen.

Die Fallberichte zu MRE in Tierkliniken konzentrieren sich vor allem auf MRE, die auch in der Humanmedizin häufig auftreten: ESBL-bildende *E.-coli (ESBL* *=* *Extended Spectrum Beta-Lactamase), A. baumannii* und MRSA. Die Möglichkeit der wechselseitigen Übertragung zwischen Mensch und Tier ist ein wesentlicher Faktor für die Verbreitung von MRE in diesen Einrichtungen [38]. Schon bei Klinikaufnahme können Haustiere mit MRE besiedelt sein, für Pferde in Deutschland werden beispielsweise Kolonisationsraten von ca. 3 % für MRSA [39] und 10 % für ESBL-*E.-coli* [40] berichtet. Während des Klinikaufenthaltes steigen diese Nachweisraten erheblich an, z. B. auf über 55 % für ESBL-*E.-coli* binnen 10 Tagen [40]. Auch wenn es erhebliche Bemühungen zur Verbesserung der Hygiene und Biosicherheit für Mitarbeitende, Tierhaltende und Tiere gibt [41], wird die Umsetzung entsprechender Empfehlungen oftmals als ungenügend eingestuft [42].

MRSA-Infektionen sowie Ausbrüche in Kleintier- und Pferdekliniken werden weltweit beschrieben [38]. Die dabei auftretenden MRSA-Linien sind aus der Humanmedizin bekannt [43], bei Pferden dominiert europaweit die Linie ST398, bekannt auch als „livestock-associated MRSA“ [44, 45]. Die Anpassung verschiedener MRSA-Linien an multiple Wirtsspezies wird u. a. durch Aufnahme von Faktoren für die Immunevasion ermöglicht [46]. Die wechselseitige Übertragung von MRSA zwischen Mensch und Tier kann dazu führen, dass Dekolonisierungserfolge bei betroffenen Patienten ausbleiben, so lange Haustiere kolonisiert sind [47].

International erfolgreiche, in der Humanmedizin als „high-risk epidemic lineages“ beschriebene extraintestinale *E.-coli*-Linien wie ST131, ST10, ST641, ST648 treten auch bei Haustieren und in Tierkliniken auf [40, 48–52]. Während für Hunde die Fütterung von kontaminiertem Rohfleisch [53] signifikant mit ESBL-*E.-coli-*Kolonisierung assoziiert ist [54], könnte für Pflanzenfresser wie Pferde eine endophytische Lebensweise von ESBL-*E.-coli *eine Rolle spielen [55]. Da viele ESBL-*E.-coli *über Abwässer, Dung und Gülle in die Umwelt gelangen, tragen diese dazu bei, dass Oberflächengewässer mit MRE belastet werden und potenzielle Reservoire für diese Erreger sein können [56–58]. Dies trägt auch zur Belastung von Wildtieren mit solchen Erregern bei.

Obwohl es für Carbapeneme keine Anwendungszulassung für Tiere insgesamt gibt, werden Fälle von Kolonisierung und Infektion durch Carbapenem-resistente Enterobakterien (CRE) bzw. Carbapenemase-produzierende Enterobakterien (CPE) bei Haustieren sporadisch berichtet. Die dabei gefundenen Isolate besitzen vor allem NDM-5- und OXA-48-Carbapenemasen [59, 60]. Auch eine nosokomiale Verbreitung dieser Erreger in Tierkliniken ist möglich [61].

## Besiedlungen mit MRE bei Personen mit Kontakt zu Nutztieren, in der Allgemeinbevölkerung und im Gesundheitswesen

Screeninguntersuchungen in definierten Populationen erlauben die Abschätzung der Verbreitung einer asymptomatischen Besiedlung mit MRE. Die Besiedlungsraten unterscheiden sich dabei regional und in Abhängigkeit von den Testsituationen.

Bei Untersuchungen zum Vorkommen von MRE-Besiedlungen in Deutschland wurde festgestellt, dass MRSA etwa 0,7–1,3 % der Menschen in der Allgemeinbevölkerung kolonisieren [62, 63]. Bei Aufnahme in verschiedene klinische Bereiche von Krankenhäusern sind 0,2–1,7 % der Patienten nasal besiedelt [64–67]. Bei Aufnahme in Rehabilitationseinrichtungen (wo Patienten mit einem vorangegangenen Krankenhausaufenthalt übernommen werden) liegt die Rate der MRSA-Besiedlungen höher (1,8–7 %; [68, 69]) ebenso bei Bewohnern von Altenheimen und Pflegeeinrichtungen (5,5–7,6 %; [70–72]).

Die VRE-Besiedlungsraten variieren regional sehr stark und liegen zwischen 0,1 % und 1,2 % bei Krankenhausaufnahme [67, 73], zwischen 2,4 % und 6,1 % bei Aufnahme in Intensivpflegebereiche [66, 74, 75] und zwischen 2,3 % und 27,5 % bei (onkologischen) Hochrisikogruppen [64, 76, 77].

Besiedlungen durch ESBL-bildende Enterobakterien werden bei 5,5–8,4 % der Menschen in der Allgemeinbevölkerung nachgewiesen [78–80]. Ähnliche Kolonisierungsraten (2,6–12,7 %) werden bei Aufnahme auf Krankenhausstationen gefunden [64–66, 81–84], wobei bei Aufnahme in Risikobereiche auch über eine häufigere Besiedlung (17,5 %) berichtet wird [76]. Dabei dominieren ESBL-*E.-coli.* Insgesamt 42–47 % davon sind auch Ciprofloxacin-resistent und gehören damit zu den 3MRGN [79, 83].

Die Besiedlung mit CRE liegt in Untersuchungen aus Deutschland bei < 0,2 % in verschiedenen Testsituationen bei Krankenhausaufnahme, ambulanten Patienten, in der Pädiatrie und auf Intensivstationen [64, 66, 67, 83, 85].

Bei einigen Bevölkerungsgruppen besteht ein erhöhtes Risiko für die Besiedlung mit MRE. Hierzu gehören Personen mit beruflicher Exposition gegenüber (Nutz‑)Tieren. Sie weisen häufiger als die Allgemeinbevölkerung Besiedlungen durch (Livestock-assoziierte) MRSA oder ESBL-*E.-coli* auf. Dagegen entsprechen die Kolonisierungsraten bei Mitarbeitenden im Gesundheitswesen denen in der Allgemeinbevölkerung bzw. denen der Patienten (Tab. 4).

**Table 4:** 

Erreger	Anzahl Isolate (*n*)	Setting	Zeitraum [Referenz]	Besiedlung (%)
**Berufe mit Tierexpositionen**				
MRSA	113	Schweinehaltung	2007–09 [86]	85,8
	59	Putenhaltung	2009 [87]	37,3
	190	Nutztierexponierte	2009–10 [88]	24,2
	35	Schweinehaltung	2010 [89]	77,1
	78	Nutztierexponierte	2012 [90]	25,6
	86	Schweinehaltung	2012 [91]	48,8
	85	Schweinehaltung	2014 [92]	84,7
	55	Kleintierklinik	2012 [93]	9,1
ESBL-*E.-coli*	73	Nutztierexponierte	2012 [94]	6,8
	86	Schweinehaltung	2012 [91]	2,5
	85	Schweinehaltung	2014 [92]	6,0
	99	Geflügelschlachthöfe	2017 [95]	5,1
**Mitarbeitende Gesundheitswesen**				
MRSA	759	Altenheim	2013 [72]	1,6
	107	Krankenhaus	2013–14 [96]	0
	83	Krankenhaus (Ärzte)	2013–15 [97]	1,2
	514	Krankenhaus (Pflege)	2013–15 [97]	5,6
	149	Krankenhaus	2014–15 [98]	0,7
	579	Ambulante Pflege	2015–16 [99]	1,2
	485	Zahnarztpraxis	2016 [100]	0
	222	Krankentransport	2016 [101]	3,2
	1005	Rehakliniken	2016–18 [102]	0,4
VRE	107	Krankenhaus	2013–14 [96]	0
ESBL-*E.-coli*	107	Krankenhaus	2013–14 [96]	3,7

*ESBL* Extended Spectrum Beta-Lactamase, *MRE* multiresistente(r) Erreger, *MRSA* Methicillin-resistente(r) *Staphylococcus aureus, VRE* Vancomycin-resistente(r) *Enterococcus (faecium)*

## Resistenzen gegen Reserveantibiotika am Beispiel der Oxazolidinonresistenz

Oxazolidinone (z. B. Linezolid) sind synthetische antimikrobielle Wirkstoffe, die ausschließlich für die humanmedizinische Anwendung zugelassen sind. Oxazolidinonresistenz kann auf chromosomalen Mutationen beruhen, hauptsächlich lokalisiert in der 23S rRNA und/oder den Genen für die ribosomalen Proteine L3 und L4, aber auch auf mobilen Resistenzgenen, die für die Methyltransferase Cfr bzw. analoge Proteine, wie Cfr(B), Cfr(C), Cfr(D) oder Cfr(E), oder aber die ABC-F-Proteine OptrA, PoxtA oder PoxtA2 kodieren. Alle bislang bekannten mobilen Oxazolidinonresistenzgene vermitteln auch Resistenz gegenüber Phenicolen. Die Cfr-Methyltransferasen vermitteln zudem Resistenz gegenüber Lincosamiden, Pleuromutilinen und Streptogramin-A-Antibiotika [103, 104].

Während chromosomale Mutationen nur vertikal verbreitet werden, spielen mobile Oxazolidinonresistenzgene eine wichtige Rolle bei der horizontalen Verbreitung entsprechender Resistenzeigenschaften. Die meisten der bislang bekannten 8 Oxazolidinonresistenzgene wurden bei unterschiedlichen Bakterien von Menschen, Tieren, Lebensmitteln tierischer Herkunft und Umweltproben gefunden. Während das Gen *cfr* initial bei *Staphylococcus* (jetzt: *Mammaliicoccus*) *sciuri* von Tieren in Deutschland nachgewiesen wurde, ist seine Präsenz mittlerweile bei Bakterien der grampositiven Genera *Staphylococcus, Streptococcus, Enterococcus, Bacillus, Jeotgalicoccus, Macrococcus *und *Mammaliicoccus*, aber auch solchen der gramnegativen Genera *Escherichia, Proteus, Providencia, Morganella, Pasteurella, Leclercia* und *Vibrio* belegt. Die entsprechenden Isolate stammten aus mindestens 25 verschiedenen Ländern von 5 Kontinenten [103, 104]. Die Varianten *cfr*(B), *cfr*(C), *cfr*(D) und *cfr*(E) sind deutlich weniger weitverbreitet, sowohl hinsichtlich ihrer geografischen Herkunft als auch ihrer Wirtsbakterien [103, 104]. Das Gen *optrA* vermittelt im Gegensatz zu allen anderen Oxazolidinonresistenzgenen auch Resistenz gegenüber Tedizolid. Dieses Gen wurde ursprünglich bei *Enterococcus* spp. von Menschen und Tieren in China gefunden, ist mittlerweile aber auch bei Vertretern der Genera *Staphylococcus, Streptococcus, Clostridium, Listeria, Lactococcus, Aerococcus* und* Vagococcus*, aber auch *Campylobacter, Fusobacterium* und *Salmonella* nachgewiesen worden. Die entsprechenden Bakterien stammten aus mindestens 29 Ländern von 6 Kontinenten [103, 104]. Das Gen *poxtA* wurde erstmalig bei einem MRSA-Isolat humanen Ursprungs in Italien gefunden und bislang ausschließlich bei grampositiven Bakterien der Genera *Staphylococcus, Enterococcus* und *Lactobacillus* in mindestens 11 Ländern und 4 Kontinenten nachgewiesen [103, 104]. Das Gen *poxtA2*, das in *Enterococcus*-Isolaten von Schweinen und Menschen nachgewiesen wurde, stellt möglicherweise einen Vorläufer von *poxtA* dar.

Die unterschiedlichen mobilen Oxazolidinonresistenzgene sind auf einer Vielzahl mobiler, genetischer Elemente in den unterschiedlichen Wirtsbakterien lokalisiert. Plasmide spielen weltweit als Träger von Oxazolidinonresistenzgenen die wichtigste Rolle. Außerdem sind Transposons, Integrative und konjugative Elemente (ICEs), mobile genomische Inseln und Prophagen an der Verbreitung der Oxazolidinonresistenzgene beteiligt [103, 104]. Viele dieser mobilen genetischen Elemente tragen zusätzliche Gene, die Resistenz gegenüber anderen antimikrobiellen Wirkstoffen, Metallen oder Bioziden vermitteln und somit die Co-Selektion von Oxazolidinonresistenzgenen selbst in Abwesenheit eines direkten Selektionsdrucks durch die Anwendung von Oxazolidinonen oder Phenicolen begünstigen [103, 104].

Nur wenige Daten liegen zur Häufigkeit von Oxazolidinonresistenzen bei Isolaten von Tieren aus Deutschland vor. Im Rahmen der Tätigkeit des „Nationalen Referenzlabors für koagulasepositive Staphylokokken“ (einschließlich *Staphylococcus aureus*; NRL-Staph) wiesen nur 2 von 4000 (0,05 %) untersuchten MRSA-Isolaten aus Tieren und Lebensmitteln eine Resistenz gegen Linezolid auf [105]. Von den im Zeitraum 2015 bis 2022 im Zoonosen-Monitoring untersuchten 2002 Enterokokkenisolaten von Masthühnern, Mastputen, Mastschweinen und Jungrindern war kein Isolat Linezolid-resistent (Daten aggregiert aus dem Zoonosen-Monitoring 2015–2021).

## MRE und Antibiotikaresistenzgene in Umweltkompartimenten

Bakterien der Kolonisationsflora von Menschen und Tieren sind in unterschiedlicher Weise zum Überleben in der Umwelt befähigt. Bestimmte Gruppen, etwa die Nonfermenter (z. B. Pseudomonas, Acinetobacter), kommen bevorzugt in der Umwelt vor.

Zum Auftreten von MRE und antimikrobiellen Resistenzen (AMR) in Umweltkompartimenten liegen bislang nur sporadische Berichte und keine systematischen Monitoringergebnisse vor. In einem Review aus dem Jahr 2020 zu Untersuchungen in Deutschland, Österreich und der Schweiz wurden aktuelle Studien zur Antibiotikaresistenz in der Umwelt analysiert [106]. Aus insgesamt 404 ausgewählten Studien wurden 52 geeignete Studien berücksichtigt, die die verschiedenen Umweltbereiche (Wasser, Abwasser, Tierhaltung, Wildtiere, Erdboden und Sediment) umfassen. Eingeschlossen wurden sowohl phänotypische als auch genotypische Resistenzergebnisse.

In diesem Review konnten große Unterschiede im Hinblick auf Studiendesign und Studienziel sowie Probennahme, Probennahmeort, Untersuchungstarget (Mikroorganismen und/oder deren Resistenzgene) und Empfindlichkeitstestung festgestellt werden. Hinsichtlich der Untersuchungsschwerpunkte der untersuchten bakteriellen Erreger, Anzahl und Dauer der Probennahme und in der Darstellung der Ergebnisse wurden wenige Übereinstimmungen gefunden. Zudem lag eine Repräsentativität der Datenerhebungen nicht vor. In keiner der eingeschlossenen Studien wurde eine epidemiologische Untersuchung vorgenommen, bei der die Verbreitung von Antibiotikaresistenzen in Verbindung zur Prävalenz mit der Bevölkerung stand. Zudem wurden keine Untersuchungen simultan in den 3 Bereichen Mensch, Tier und Umwelt vorgenommen.

Am häufigsten wurden Proben aus Abwasseraufbereitungsanlagen untersucht, da sie als Hotspot für Antibiotikaresistenzen gelten und bei Verwendung von Klärschlamm als Dünger Antibiotikaresistenzen oder Antibiotikaresistenzgene in Futtermittel und die Lebensmittelkette eingetragen werden können [107]. Es wurden Resistenzen gegenüber allen Antibiotika beschrieben, die in der „WHO-CIA-Liste“ („list of critically important antimicrobials for human medicine“, s. oben) zu finden sind. Resistenzen gegenüber antimikrobiellen Wirkstoffen aus den Kategorien Critically Important und Highly Important wurden am häufigsten aufgeführt. In den meisten Studien wurden *E. coli* und coliforme Bakterien isoliert.

Klees et al. (2020) beschreiben den kulturellen Nachweis von CPE und VRE in der Umwelt (Wasser, Abwasser, Boden, Klärschlamm) und zudem den PCR-basierten Nachweis von Resistenzgenen, die für Carbapenemasen (*bla*_OXA-48_, *bla*_KPC_, *bla*_VIM/NDM_) kodieren ohne bestätigende Kultivierung [108]. Zudem wurden Colistinresistenzen, das *mcr-1-*Gen und ESBL-*E.-coli* in der Umwelt gefunden.

Eine Studie aus Deutschland [109] untersuchte Abwasser und Prozesswasser eines Geflügelschlachthofes auf ESKAPE-Bakterien (*Enterococcus spp., S. aureus, K. pneumoniae, A. baumannii, P. aeruginosa, Enterobacter spp.*) und *E. coli *und konnte dabei die Resistenzen sowohl phänotypisch als auch genotypisch sowie verschiedene Beta-Laktamase‑, Carbapenemase- und mobile Colistinresistenzgene bestimmen. Das Abwasser enthielt *E. coli*, MRSA, *K. pneumoniae* und Spezies der *A.-baumannii-* und *Enterobacter-cloacae*-Komplexe mit klinisch relevanten Resistenzgenen, wie z. B. *bla*_CTX‑M_ oder *bla*_SHV_ und *mcr‑1*.

Eine weitere Studie [110] untersuchte das Abwasser deutscher Krankenhäuser und somit einen möglichen Eintrag von CPE in die Umwelt. Es wurden sowohl Bakterien mit Resistenzen gegen Cephalosporine der 3. Generation als auch Isolate mit den Carbapenemasegenen *bla*_VIM_, *bla*_NDM_ und *bla*_OXA-48_ bzw. der Definition „XDR“ (Stämme mit einer Empfindlichkeit gegenüber ein oder 2 antimikrobiellen Substanzen) isoliert. Auch wenn die meisten Bakterien während der Abwasseraufbereitung eliminiert werden, ist ein Eintrag einzelner Resistenzgene in das Wasser und somit in die Umwelt möglich. Auch der Nachweis von ESBL-*E.-coli *in Abwasserproben aus privaten Haushalten bestätigt die Annahme, dass eine Verbreitung von Resistenzgenen über das Abwasser grundsätzlich möglich ist. Insbesondere sozial benachteiligte Stadtteile und die Zeit der Wintermonate zeigten eine höhere Last an resistenten Bakterien im Abwasser. Bei knapp 30 % der Bakterien handelte es sich um multiresistente gramnegative Erreger [111].

Weitere Untersuchungen sind erforderlich, insbesondere unter Anwendung harmonisierter Methoden zur Gewinnung und Empfindlichkeitstestung von bakteriellen Erregern, zur Definition einzubeziehender Umweltkompartimente und deren adäquater Beprobung sowie zur Analyse möglicher Übertragungswege. Um den Umweltfaktor bezüglich der Verbreitung von Resistenzen besser erfassen zu können, stellen diese eine nicht unerhebliche Herausforderung dar. Des Weiteren könnte eine Ausweitung des „WHO-Tricycle-Protokolls“^7^ auf andere Antibiotikaresistenzindikatoren spezifisch für ein bestimmtes Reservoir in der Umwelt vorteilhaft sein. Rückstände und Abbauprodukte antimikrobieller Substanzen in Umweltkompartimenten spielen ebenfalls eine Rolle bei der Selektion und Anreicherung von MRE. Wir konnten aus Kapazitätsgründen dieses auch wichtige Thema nicht aufgreifen und verweisen auf entsprechende Artikel [112–114].

## Herausforderungen bei der Bewertung der gesamten Resistenzsituation im One-Health-Kontext

Insgesamt lassen sich die bislang dokumentierten Ergebnisse auf eine Vielzahl von Untersuchungen aus verschiedenen Sektoren zurückführen. Um diese Erkenntnisse einer Gesamtbewertung zu unterziehen, sollten aus epidemiologischer Sicht die Datenquellen der Untersuchungsergebnisse mitberücksichtigt werden.

Grundsätzlich kann man diese Quellen unterscheiden in Untersuchungen (i) im Rahmen der Diagnostik, (ii) zur vertiefenden Differenzierung sowie (iii) zum Screening oder zur eigenständigen Durchführung epidemiologischer Studien nach definierten Studiendesigns. Insbesondere die Ergebnisse aus (i) und (ii) sind einem gewissen Selektionseffekt unterworfen, d. h., sie stellen nur ausgewählte Patientenkollektive dar, so dass die gefundenen Anteile resistenter Isolate nicht für die allgemeine Bevölkerung, sondern stets für spezifische Populationen stehen. So werden beispielsweise Patienten ggf. erst dann diagnostischen Untersuchungen unterzogen, wenn bei gängigen Therapien mit Antibiotika keine Wirkung beobachtet wurde. Dementsprechend liegen hier die Antibiotikaresistenzraten höher als in der gesunden Population (z. B. bei Erregern von Harnwegsinfektionen).

Demgegenüber bieten die Untersuchungen zu (iii) je nach Studiendesign die Möglichkeit, repräsentative Aussagen zur Allgemeinbevölkerung oder zu bestimmten Bevölkerungsgruppen zu treffen bis hin zu solchen mit Bezug zum One-Health-Kontext (gemeinsame Auswertung von Proben aus Humanmedizin, Veterinärwesen, Landwirtschaft und Umwelt). Daten aus dem Lebensmittelmonitoring werden in regelmäßiger Routine von den zuständigen Behörden erhoben und unterliegen festgelegten Dokumentationsrichtlinien. Diese Daten sind deshalb als „annähernd zufällig ausgewählt“ anzusehen und besitzen zudem gut dokumentierte Metadaten.

Bis auf wenige Ausnahmen systematischer epidemiologischer Studien [115–117] sind die meisten Untersuchungen auf bereits bestehende Datenquellen ausgerichtet und können daher allgemein dem Bereich der Sekundärdatenanalyse bzw. der Versorgungsforschung zugeordnet werden [118]. Daher gelten auch für diese Studien die typischen Restriktionen, dass vor einer spezifischen Auswertung zunächst die Prüfung der Daten für diesen Untersuchungszweck erfolgen muss. Als typisches Beispiel kann hier der Aufbau einer „Genombasierten Surveillance übertragbarer Colistin- und Carbapenemresistenzen Gram-negativer Infektionserreger“ (Forschungsverbund GÜCCI) angesehen werden.^8^ Datenquellen sind hierbei Informationen aus allen Segmenten (i), (ii) und (iii). Sollen diese genutzt werden, sind dann neben der Definition des Auswertungszweckes eine erhebliche harmonisierende Nachbearbeitung der Datenstrukturen, ggf. eine spezifische Selektion von Isolaten und das Hinzuziehen von Metainformationen zu den Proben (beispielweise aus den Patientenakten) erforderlich. Da genau diese Daten häufig in den Laborinformationssystemen fehlen und zudem Datenschutzfragen die Nutzung derzeit einschränken, sind hier weitergehende fachliche wie juristische Bewertungen dringend erforderlich, bevor eine finale Bewertung der Resistenzsituation hinsichtlich der Schnittmenge erfolgen kann.

Ein Protokoll zur Harmonisierung der Probennahme, des Probenentnahmeortes und der Empfindlichkeitstestung ist maßgebend, um Ergebnisse untereinander und mit allen One-Health-Bereichen Mensch – Tier – Umwelt vergleichen zu können. Notwendig sind auch systematisch durchgeführte epidemiologische Studien zur Untersuchung von Antibiotikaresistenzen unter Berücksichtigung der verschiedenen One-Health-Bereiche.

## Fazit

Zur Problematik von AMR und MRE sowie zum Antibiotikaverbrauch liegen in Deutschland umfangreiche Daten aus der Human- und Veterinärmedizin vor. Diese Informationen werden in unterschiedlichen Kontexten nach verschiedenen Vorgaben und Standards erhoben, was den Vergleich der Resultate erschwert. Es gibt weiterhin erheblichen Harmonisierungsbedarf bezüglich der untersuchten Probenkollektive, der verwendeten Standards und analysierten Datenformate (inkl. Ganzgenomdaten).

In einzelnen Bereichen sind die Anstrengungen zur Harmonisierung auf einem guten Weg, z. B. im Bereich der sektorübergreifenden Anpassung der antimikrobiellen Empfindlichkeitstestung^9^ [119] und bei Bestrebungen zur Standardisierung der genombasierten Erreger- und AMR-Surveillance [120]. Zu einem gewissen Anteil liegen auch Ergebnisse aus sektorübergreifenden Studien vor, in welchen die Daten nach einem abgestimmten Schema und vorgegebenen Standards erhoben wurden [121–123]. Dabei wird deutlich, dass eine detaillierte epidemiologische und molekulare Analyse notwendig ist, um die genaue Größe von Schnittmengen abzuschätzen [13, 122, 124, 125]. Neben einer Harmonisierung der Methoden müssen auch stets Interpretationsregeln für den One-Health-Kontext bereitgestellt werden.

Zu Resistenzen gegen humanmedizinische Reservesubstanzen wie Colistin und Linezolid ist die Ausgangsbasis an vorliegenden Daten sehr heterogen. Bei Colistin weisen aktuelle Zahlen aus der Routinesurveillance bei Nutztieren und Lebensmitteln eine weite Verbreitung *mcr-*vermittelter Colistinresistenz in *E.-coli-*Isolaten aus. Im humanen Bereich wird Colistinresistenz eher selten untersucht. Wenn sie in seltenen Fällen nachgewiesen wird, ist sie eher chromosomal determiniert (Forschungsverbund GÜCCI; unpublizierte Daten). Linezolidresistenzen sind in allen Sektoren selten [105, 126]; Plasmid-vermittelte Resistenzen scheinen hier eine gewisse Rolle zu spielen und können zwischen Isolaten verschiedener Gattungen und Sektoren ausgetauscht werden [103]. Carbapenemresistenzen sind nach wie vor in Deutschland selten und vor allem bei pathogenen Erregern vom Menschen nachweisbar.

Der Zusammenhang zwischen einem Einsatz von antimikrobiellen Wirkstoffen und einer direkten oder indirekten Selektion von MRE ist gut dokumentiert. Erfolge in der Minimierung des Einsatzes moderner Antibiotika bzw. von Substanzen mit einem humanmedizinischen Vorbehalt konnten in zurückliegenden Jahren dargestellt werden [127–129]. Auf eine Reduktion des Einsatzes bestimmter Antiinfektiva folgt nicht zwangsläufig und unmittelbar eine Reduktion der Resistenzrate gegenüber dieser Substanz [130–133]. Bestrebungen von Antibiotic-Stewardship-Teams führten zur Reduktion des Einsatzes von Fluorchinolonen und modernen Cephalosporinen im stationären Bereich; gemeinsam mit weiteren Bestrebungen zur Verbesserung der Infektionsprävention (z. B. Screening, Einzelunterbringung, Dekolonisierung) wird dies mit den sinkenden Raten der Oxacillinresistenz bei *S. aureus* und rückläufigen Meldungen von MRSA aus Blutstrominfektionen in Verbindung gebracht. Im selben Zeitraum stiegen jedoch die Raten der Vancomycinresistenz bei *E. faecium* in deutschen Krankenhäusern deutlich an, wobei das gesteigerte Auftreten von VRE in vielen Studien mit einem verstärkten Einsatz von Fluorchinolonen und modernen Cephalosporinen assoziiert ist [128]. Bei Enterokokken gibt es keine Belege für eine Verbreitung der Vancomycinresistenz in Isolaten aus Nutztieren und/oder Lebensmitteln in Deutschland.

Es ist offensichtlich und unbestritten, dass AMR und MRE in einem übergeordneten One-Health-Kontext betrachtet werden müssen. Jedoch weisen nicht alle Resistenzprobleme im humanen und im veterinärmedizinischen Bereich eine Verknüpfung mit anderen Sektoren auf; weswegen sektorspezifische Maßnahmen zur Senkung der Last durch MRE und AMR ebenso notwendig sind.

## Footnotes

1 https://www.rki.de/DE/Content/Infekt/Jahrbuch/jahrbuch_node.html (letzter Zugriff 27.04.2023).

2 https://www.nrz-hygiene.de/KISS-Modul/referenzdaten/KISS/ITS-KISS-Erreger-Surveillance (letzter Zugriff 27.04.2023).

3 3MRGN: Resistenz gegen Piperazillin, Ciprofloxacin und einem 3. Generation-Cephalosporin (für die genauen Definitionen, s. Bundesgesundheitsblatt 2012; 55: 1311–1354).

4 4MRGN: 3MRGN und zusätzliche Carbapenemresistenz (für die genauen Definitionen, s. Bundesgesundheitsblatt 2012; 55: 1311–1354).

5 DDD: „defined daily dose“, dt.: angenommene mittlere Tagesdosis.

6 https://avs.rki.de/Content/ReferenceData/HospitalComparisonTime.aspx (letzter Zugriff 27.04.2023).

7 https://www.who.int/publications/i/item/9789240021402.

8 https://www.rki.de/DE/Content/Institut/OrgEinheiten/Abt1/FG13/guecci.html.

9 https://www.eucast.org/organization/subcommittees/vetcast.
